# The Principles of Physiology Applied to the Preservation of Health and to the Improvement of Physical and Mental Education

**Published:** 1841-10

**Authors:** 


Art. III.—*The Principles of Physiology applied to the Preservation of Health and to the Improvement of Physical and Mental Education.* By Andrew Combe, m.d. &c. *With Thirteen Woodcuts.* Tenth Edition, revised and enlarged.—*Edinburgh*, 1841. 8vo, pp. 420.

In our Second Number for April, 1836, we noticed, at some length, the Third Edition of this work, and bestowed on it that high and unqualified praise which can only belong to productions of the very first order. It is unnecessary to go over the same ground again, but we feel it due to such of our present readers as may not have seen our former article, to call their attention to a treatise which contains more sound philosophy, more true practical wisdom, relative to the all-important subject of preserving health, than any other volume in our language; and which, while it is calculated to please and benefit the public generally, is no less adapted for the study of professional men. The extremely large sale of the former editions of this work is, at once, evidence of its merits, and a gratifying proof that the reading part of the public are becoming daily more awake to the importance of that branch of philosophy of which it treats. Since its first appearance in the spring of 1834, "nine editions, consisting together of 14,000 copies have been exhausted in this country, and upwards of 30,000 copies in the United States of America." We believe such a wide circulation as this of a medical book, can only be stated of that before us and of the other works of Dr. Combe.

